# Adipose transplantation improves metabolism and atherosclerosis but not perivascular adipose tissue abnormality or vascular dysfunction in lipodystrophic *Seipin/Apoe* null mice

**DOI:** 10.1152/ajpcell.00698.2023

**Published:** 2024-03-25

**Authors:** Zhe Meng, Chuangxing Liu, Mengke Xu, Yongqiang Tao, Haiyu Li, Xijia Wang, Jiawei Liao, Mengyu Wang

**Affiliations:** ^1^Department of Cardiology, First Affiliated Hospital of Zhengzhou University, Zhengzhou, Henan, China; ^2^Institute of Cardiovascular Diseases, First Affiliated Hospital of Dalian Medical University, Dalian, Liaoning, China

**Keywords:** adipose transplantation, atherosclerosis, lipodystrophy, metabolism, SEIPIN

## Abstract

Adipose dysfunction in lipodystrophic SEIPIN deficiency is associated with multiple metabolic disorders and increased risks of developing cardiovascular diseases, such as atherosclerosis, cardiac hypertrophy, and heart failure. Recently, adipose transplantation has been found to correct adipose dysfunction and metabolic disorders in lipodystrophic *Seipin* knockout mice; however, whether adipose transplantation could improve lipodystrophy-associated cardiovascular consequences is still unclear. Here, we aimed to explore the effects of adipose transplantation on lipodystrophy-associated metabolic cardiovascular diseases in *Seipin* knockout mice crossed into atherosclerosis-prone apolipoprotein E (*Apoe*) knockout background. At 2 months of age, lipodystrophic *Seipin/Apoe* double knockout mice and nonlipodystrophic *Apoe* knockout controls were subjected to adipose transplantation or sham operation. Seven months later, mice were euthanized. Our data showed that although adipose transplantation had no significant impact on endogenous adipose atrophy or gene expression, it remarkably increased plasma leptin but not adiponectin concentration in *Seipin/Apoe* double knockout mice. This led to significantly reduced hyperlipidemia, hepatic steatosis, and insulin resistance in *Seipin/Apoe* double knockout mice. Consequently, atherosclerosis burden, intraplaque macrophage infiltration, and aortic inflammatory gene expression were all attenuated in *Seipin/Apoe* double knockout mice with adipose transplantation. However, adipocyte morphology, macrophage infiltration, or fibrosis of the perivascular adipose tissue was not altered in *Seipin/Apoe* double knockout mice with adipose transplantation, followed by no significant improvement of vasoconstriction or relaxation. In conclusion, we demonstrate that adipose transplantation could alleviate lipodystrophy-associated metabolic disorders and atherosclerosis but has an almost null impact on perivascular adipose abnormality or vascular dysfunction in lipodystrophic *Seipin/Apoe* double knockout mice.

**NEW & NOTEWORTHY** Adipose transplantation (AT) reverses multiply metabolic derangements in lipodystrophy, but whether it could improve lipodystrophy-related cardiovascular consequences is unknown. Here, using *Seipin/Apoe* double knockout mice as a lipodystrophy disease model, we showed that AT partially restored adipose functionality, which translated into significantly reduced atherosclerosis. However, AT was incapable of reversing perivascular adipose abnormality or vascular dysfunction. The current study provides preliminary experimental evidence on the therapeutic potential of AT on lipodystrophy-related metabolic cardiovascular diseases.

## Graphical abstract

**Figure:**
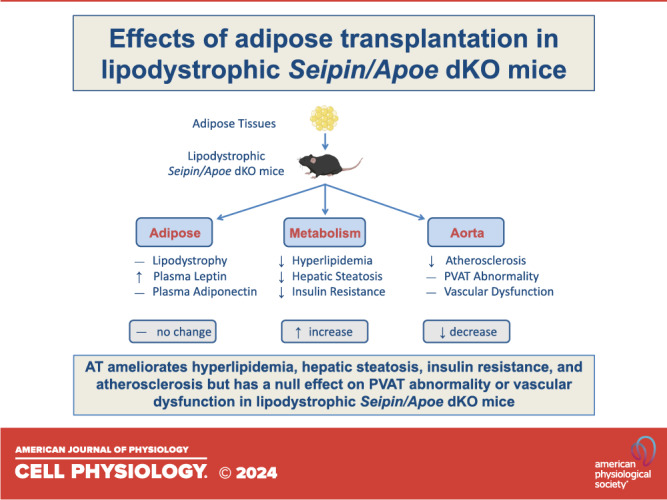

## INTRODUCTION

The adipose tissue is a dynamic organ with remarkable expandability and metabolic flexibility in response to both external and internal stimuli, including nutritional status, aging, physical activity, drug consumption, and disease conditions (1). It is highly heterogeneous and can be classified into fat-storing white adipose tissue (WAT), thermogenic brown adipose tissue (BAT), and a middle type, beige/brite adipose, which comprises inducible brown-like adipocytes residing in WAT depots (2). Moreover, it also serves as an important endocrine organ that is capable of secreting a wide range of metabolism-active (such as leptin and adiponectin) and inflammation-associated (such as IL-6 and TNF-α) adipokines (3). Therefore, the adipose plays an essential role in maintaining systemic metabolic homeostasis and cardiovascular health.

With the rapid growth of the obese population, increased adiposity in obesity has become a major global concern in the past half-century. Numerous studies have demonstrated that obesity is a major risk factor for metabolic disorders and cardiovascular diseases (4, 5). In recent years, however, insufficient adiposity in lipodystrophy is gaining more and more attention. Depending on the etiology, lipodystrophy can be divided into two types: congenital lipodystrophy caused by genetic defects, and acquired lipodystrophy seen in certain diseases, such as measles, systemic lupus erythematosus, and dermatomyositis, or induced by certain medications such as highly active antiretroviral therapy (HAART) in human immunodeficiency virus-infected patients (6). The former congenital lipodystrophy can be further classified into congenital generalized lipodystrophy [CGL; also known as Berardinelli-Seip congenital lipodystrophy (BSCL)] and familial partial lipodystrophy, based on the degree of fat loss, while the latter acquired lipodystrophy can be further divided into acquired generalized lipodystrophy (also known as Lawrence syndrome), acquired partial lipodystrophy (also known as Barraquer-Simons syndrome), and HAART-associated lipodystrophy syndrome (6).

SEIPIN, encoded by the homonym gene *Seipin* or *Bscl2,* is a membrane protein located in the endoplasmic reticulum. It is known as the culprit for human CGL2/BSCL2, the most severe form of human lipodystrophy (7, 8). Predominantly expressed in the adipose, brain, and testis, *Seipin* can be found with lower expression in other organs such as the liver, kidney, heart, and aorta (8, 9). The main function of SEIPIN is to regulate adipocyte differentiation, adipogenesis, and lipid droplet formation (10–13). In mice, *Seipin* ablation causes a nearly complete loss of WAT and severe adipose dysfunction, followed by multiple metabolic disorders such as dyslipidemia, hepatic steatosis, insulin resistance, and glucose intolerance, eventually resulting in an increased risk of developing cardiovascular diseases including atherosclerosis (14, 15), diabetic cardiomyopathy (16), cardiac hypertrophy, and heart failure (17, 18). Of note, transplantation of functional adipose tissues is sufficient to repair adipose dysfunction and improve lipodystrophy-associated metabolism and renal injury in *Seipin* knockout (KO) mice (19, 20). However, it is still unknown whether adipose transplantation (AT) can improve lipodystrophy-associated metabolic cardiovascular diseases in *Seipin* deficiency. In this study, we aimed to explore this issue with a focus on vasculopathy in *Seipin* KO mice crossed into an atherosclerosis-prone apolipoprotein E (*Apoe*) KO background.

## MATERIALS AND METHODS

### Animals

*Seipin* KO mice (C57BL/6J background) were generated as previously described (21) and crossed with *Apoe* KO mice (eKO; C57BL/6J background, purchased from Beijing Vital River Laboratory Animal Technology Co., Ltd.) to generate *Seipin/Apoe* double (d)KO mice (15). Only male *Seipin/Apoe* dKO mice (*n* = 16) and matched *Apoe* KO littermates (*n* = 16) were chosen in the study and randomly and evenly divided into the AT group and the Sham group. All mice were housed at room temperature (22°C) in individually ventilated cages with a 12-h light/dark cycle and were allowed free access to a rodent chow diet and sterilized water. The animal study was approved by the Animal Care Committee of Zhengzhou University and performed under the *Guidelines for the Care and Use of Laboratory Animals* of the National Institutes of Health.

### AT Procedure

Mouse AT operation was performed as previously described (19, 20). Briefly, 2-month-old mice were anesthetized by intraperitoneal injection of 1% pentobarbital sodium (45 mg/kg body wt). Subcutaneous white adipose tissues collected from 2- to 3-month-old *Apoe* KO males were used as donor fats and divided into 100- to 150-mg fat pieces. Each recipient mouse of the AT group was transplanted with ∼900 mg donor fats (6 to 8 fat pieces) through subcutaneous incisions in the back, while mice of the Sham group underwent the same procedure without actual graft implantation. All mice were housed individually for 1 week to allow for recovery from the operation and then kept in four per cage. Seven months after the operation, all mice were humanely euthanized by CO_2_ inhalation.

### Blood Pressure and Plasma Biochemical Assay

Blood pressure was recorded by a noninvasive tail-cuff method (BP-2010; Softron, Tokyo, Japan) and averaged from ten measurements per mouse (22). For plasma biochemical analysis, mice were fasted for 4 h and then blood was collected into heparin-coated tubes by retro-orbital bleeding. Plasma total cholesterol, triglycerides, and glucose levels were determined using commercial enzymatic kits (BioSino, Beijing, China), while leptin (SEKM-0105; Solarbio, Beijing, China), adiponectin (SEKM-0142; Solarbio, Beijing, China), and insulin (SEKM-0141; Solarbio, Beijing, China) levels were determined using ELISA kits.

### Glucose and Insulin Tolerance Tests

The glucose tolerance test (GTT) and insulin tolerance test (ITT) were performed as previously described at 3 and 2 weeks before the mice were euthanized (23, 24). Briefly, after 4 hours of fasting, the mice were intraperitoneally injected with glucose (2 g/kg body wt; Abbott Laboratories) or insulin (0.75 mIU/g body wt; Eli Lilly). Blood samples were collected before (time 0) and at 15, 30, 60, and 120 (for GTT)/90 (for ITT) minutes postintraperitoneal injection for the measurement of plasma glucose level as described above.

### Hepatic Lipid and Lipid Perioxidation Analysis

After perfusion, the liver was removed and weighed. For histological analysis of hepatic lipid accumulation, the fixed liver samples were embedded in paraffin and sectioned at 5-μm thickness. Lipid vacuoles were visualized by hematoxylin and eosin (H&E) staining. For hepatic lipid quantitation, ∼100 mg of liver samples were homogenized in 1 mL phosphate buffer solution. Lipids were extracted using Folch’s reagent and dissolved in 1 mL 3% Triton X-100 (25). Triglyceride content in the solutions was then measured with commercial kits (BioSino, Beijing, China) and normalized to liver weight. The hepatic lipid perioxidation level was determined by measuring the malondialdehyde level in the liver samples using a commercial kit (BC0025; Solarbio, Beijing, China), according to the manufacturer’s guidance.

### RNA Isolation and Quantitative Real-Time PCR

RNA extraction and quantitative real-time PCR were performed as previously described (22). Briefly, total RNA was extracted using Tri-reagent (R1100; Solarbio, Beijing, China) and reverse-transcribed into cDNA with an RT kit (HY-K0510A; MedChemExpress, Monmouth Junction, NJ). Quantitative real-time PCR was performed with SYBR Green qPCR reagents (AN19L919; Life-iLab, Shanghai, China). All samples were quantified using the comparative CT method and normalized to glyceraldehyde-3-phosphate dehydrogenase (*Gapdh*) levels. The primers used in the study are listed in Table 1.

**Table 1.** List of primers used in the study

Gene	Forward	Reverse
*Leptin*	CAAGCAGTGCCTATCCAGAA	GGAATGAAGTCCAAGCCAGT
*Adiponectin*	GATGGCAGAGATGGCACTCC	CTTGCCAGTGCTGCCGTCAT
*Pparγ*	GACCACTCGCATTCCTTT	CCACAGACTCGGCACTCA
*Acc1*	CTCCCGATTCATAATTGGGTCTG	TCGACCTTGTTTTACTAGGTGC
*Fas*	GGGTCTATGCCACGATTC	GTGTCCCATGTTGGATTTG
*Scd1*	CGCTGGCACATCAACTTCAC	AGGAACTCAGAAGCCCAAAGC
*Mtp*	GGAAAGCAGAGCGGAGAC	AGAGCAAGGGTCAGGCAC
*Pparα*	GGGCTTTCGGGATAGTTG	ATTGGGCTGTTGGCTGAT
*Cpt1α*	CTCCGCCTGAGCCATGAAG	CACCAGTGATGATGCCATTCT
*Irs1*	GGATCGTCAATAGCGTAA	GCTTGGCACAATGTAGAA
*Irs2*	GGGGCGAACTCTATGGGTA	GCAGGCGTGGTTAGGGAAT
*Akt2*	CAGATGGTCGCCAACAGT	TGCCGAGGAGTTTGAGATA
*Glut4*	ACGGATAGGGAGCAGAAA	AAGGGTGAGTGAGGCATT
*Mcp-1*	TAAAAACCTGGATCGGAACCAAA	GCATTAGCTTCAGATTTACGGGT
*Il-1β*	CTTCCCCAGGGCATGTTAAG	ACCCTGAGCGACCTGTCTTG
*Il-6*	TTCCATCCAGTTGCCTTCTTG	TTGGGAGTGGTATCCTCTGTGA
*Col1a1*	CGCCATCAAGGTCTACTGC	GAATCCATCGGTCATGCTCT
*Col3a1*	GGCAGTGATGGGCAACCT	TCCCTTCGCACCGTTCTT
*Gapdh*	TGATGACATCAAGAAGGTGGTGAAG	TCCTTGGAGGCCATGTAGGCCAT

### Atherosclerosis Analysis

The entire aortas and hearts were harvested after perfusion and prepared as previously described (26). Briefly, the fixed aortas were cut open longitudinally and the adventitia was removed under a dissecting microscope. The fixed hearts were embedded in O.C.T. compound (Sakura Finetek, Torrance, CA), snap-frozen in liquid nitrogen, and cross-sectioned serially at the aortic root level at 7-μm thickness. Atherosclerotic burden in the inner surface of the entire aorta and the cross-sectioned aortic roots were visualized by Oil-red O (Sigma, St. Louis, MO) staining. Using aortic root cryosections, the plaque macrophage content was visualized by CD68 (ab53444, diluted at 1:300; Abcam, Cambridge, UK) immunochemical staining. Quantification of lesion area and CD68 positive area was determined with ImageJ software in a blinded manner.

### Perivascular Adipose Tissue Histology and Vasoactivity Analysis

The thoracic aortas were collected and placed in a cold (4°C) Krebs-Ringer bicarbonate solution (containing 118.6 mmol/L NaCl, 4.7 mmol/L KCl, 2.5 mmol/L CaCl_2_, 1.2 mmol/L MgSO_4_, 1.2 mmol/L KH_2_PO_4_, 25.1 mmol/L NaHCO_3_, 0.026 mmol/L EATANA_2_Ca, and 10.1 mmol/L glucose). Vasoactivity analysis was performed using an aortic ring experiment as previously described (9). Briefly, aortic rings (2 mm length) with perivascular adipose tissue (PVAT) were gently mounted in a myograph system and contracted with KCl. Constriction response to phenylephrine (PE; ranging from 10^−9^ to 10^−5^ mol/L, Sigma-Aldrich) was recorded and expressed as the contraction percentage of the maximum contraction to KCl. Relaxation response to acetylcholine (Ach; ranging from 10^−9^ to 10^−5^ mol/L, Sigma-Aldrich) or sodium nitroprusside (SNP; ranging from 10^−9^ to 10^−5^ mol/L, Sigma-Aldrich) was recorded after precontraction with PE and expressed as the percentage of the maximum response to Ach or SNP, respectively. The PVAT removed from the thoracic aortas was then fixed, embedded in paraffin, and sectioned at 2-μm thickness. Gross morphology of the PVAT was visualized by H&E staining, with the size of perivascular adipocytes presented as the distribution of an individual adipocyte population according to size (*n* ≥ 200 adipocytes per mouse). Macrophage content in the PVAT was visualized by CD68 (ab53444, diluted at 1:300; Abcam, Cambridge, UK) immunochemical staining. Quantification of adipocyte size and CD68-positive area was determined with ImageJ software in a blinded manner.

### Statistical Analysis

The Shapiro-Wilk test and *F* test were used to assess data normality and homogeneity of variance. The effects of genotype (G = dKO vs. eKO) and treatment (T = AT vs. Sham) and G x T interactions were evaluated by two-way ANOVA followed by Tukey’s post hoc tests. The *P* values for the main and interaction effects were displayed below each graph as appropriate. Student’s *t* tests were also used, when applicable, to determine statistical significance between two-group comparisons. All statistical analyses were performed with Prism software and data were presented as means ± SD. *P* < 0.05 was regarded as statistically significant.

## RESULTS

### AT Partially Restores Adipose Function in Lipodystrophic *Seipin/Apoe* dKO Mice

**Figure 1.**
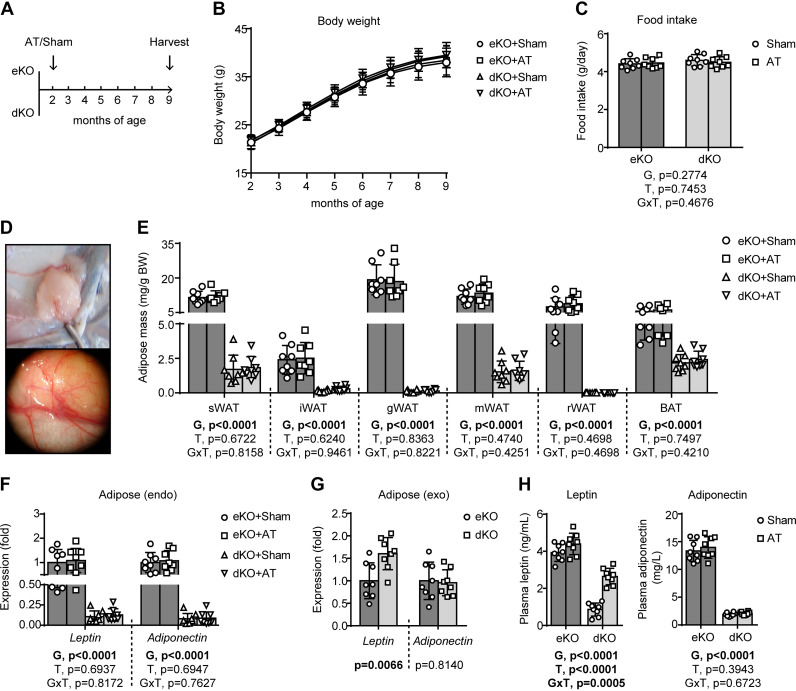
Adipose transplantation (AT) partially restores adipose function in lipodystrophic *Seipin/Apoe* double knockout (dKO) mice. *A*: schematic illustration of the experimental design. *B*: body weight during the experimental process. *C*: food intake measured before harvest. *D*: a gross view (*top*) and a photographic image (*bottom*) under a dissecting microscope showing angiogenesis of the implanted adipose in the recipient mice. *E*: mass of major fat pads, including 5 depots of white adipose tissues (WAT) and subscapular brown adipose tissues (BAT). *F*: *Leptin* and *Adiponectin* gene expression of the endogenous (endo) subcutaneous adipose. *G*: *Leptin* and *Adiponectin* gene expression of the implanted exogenous (exo) adipose. *H*: plasma leptin and adiponectin concentration. eKO, *Apoe* knockout; BW, body weight; sWAT, subcutaneous WAT; iWAT, inguinal WAT; gWAT, gonadal WAT; mWAT, mesenteric WAT; rWAT, retroperitoneal WAT. For *G*, Student’s *t* tests were used to determine statistical significance, and the *P* values are displayed below the graph; for *A*–*F* and *H*, the effects of genotype (G = dKO vs. eKO) and treatment (T = AT vs. Sham) and G × T interactions were evaluated by two-way ANOVA followed by Tukey’s post hoc analysis, and the *P* values for the main and interaction effects are displayed below each graph. Error bars represent SD; *n* = 8 per group.

To investigate the potential effects of AT, 2-month-old male lipodystrophic *Seipin/Apoe* dKO mice and matched *Apoe* KO controls were randomly divided into AT and Sham groups and were euthanized 7 months after the operation (Fig. 1*A*). During the experimental period, the body weight was monitored monthly, and no statistical significance was observed among different groups (Fig. 1*B*). Food intake measured before the mice were euthanized also showed no significant difference (Fig. 1*C*). An observation of angiogenesis in the exogenous donor fats indicates successful implantation (Fig. 1*D*). Of note, AT did not affect the volume or adipokine expression of the endogenous adipose tissues in the recipient mice (Fig. 1*E* and *F*). However, it seemed that the exogenous donor fats implanted into lipodystrophic *Seipin/Apoe* dKO mice were more activated than those implanted into nonlipodystrophic *Apoe* KO controls, as evidenced by a significant increase of *Leptin* but not *Adiponectin* gene expression in the exogenous donor fats implanted into lipodystrophic *Seipin/Apoe* dKO mice (Fig. 1*G*). As a result, lipodystrophy-induced decrease of plasma leptin but not adiponectin levels in *Seipin/Apoe* dKO mice was partially reversed by AT (Fig. 1*H*). In comparison, AT had a null impact on neither plasma leptin nor adiponectin levels in nonlipodystrophic *Apoe* KO controls (Fig. 1*H*).

### AT Alleviates Dyslipidemia and Hepatic Steatosis in Lipodystrophic *Seipin/Apoe* dKO Mice

**Figure 2.**
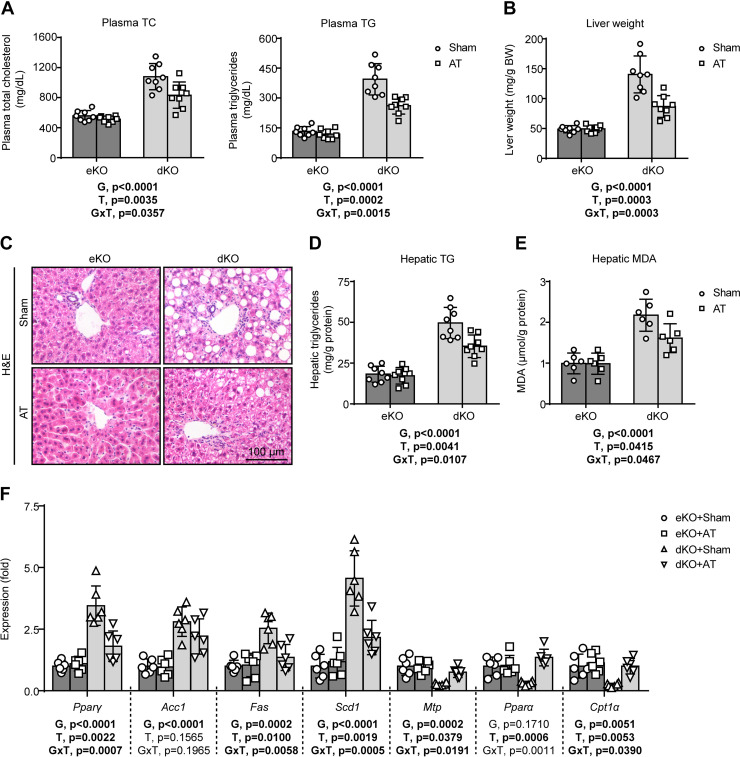
Adipose transplantation (AT) alleviates hyperlipidemia and hepatic steatosis in lipodystrophic *Seipin/Apoe* double knockout (dKO) mice. *A*: plasma total cholesterol (TC) and triglycerides (TG) concentration. *B*: liver weight (BW, body weight). *C*: representative hematoxylin and eosin (H&E) staining of liver sections. *D*: hepatic TG content. *E*: hepatic malondialdehyde concentration. *F*: hepatic lipogenesis and β-oxidation gene expression. The effects of genotype [G = dKO vs. *Apoe* knockout (eKO)] and treatment (T = AT vs. Sham) and G × T interactions were evaluated by two-way ANOVA followed by Tukey’s post hoc analysis. The *P* values for the main and interaction effects are displayed below each graph. Error bars represent SD; *n* = 6–8 per group.

Previously, we have demonstrated that lipodystrophic *Seipin/Apoe* dKO mice developed spontaneous hyperlipidemia and hepatic steatosis (15). Here we showed that AT markedly inhibited lipodystrophy-induced increase of plasma total cholesterol and triglyceride in *Seipin/Apoe* dKO mice (Fig. 2*A*). Moreover, AT significantly reduced lipodystrophy-induced increase of liver weight (Fig. 2*B*) and hepatic lipid accumulation in *Seipin/Apoe* dKO mice, as indicated by less lipid vacuoles seen in H&E staining (Fig. 2*C*) and decreased triglyceride contents using hepatic lipid extraction (Fig. 2*D*), Meanwhile, AT significantly inhibited lipodystrophy-induced increase of hepatic malondialdehyde (MDA) concentration, a marker of lipid peroxidation level, in *Seipin/Apoe* dKO mice (Fig. 2*E*). Using real-time quantitative PCR, we further showed that AT alleviated lipodystrophy-induced activation of hepatic lipogenesis (*Pparγ*, *Fas*, and *Scd1*) and inhibition of triglyceride-rich lipoprotein packaging (*Mtp*) and fatty acid β-oxidation (*Pparα* and *Cpt1α*) in *Seipin/Apoe* dKO mice (Fig. 2*F*). Notably, AT hardly affected neither plasma nor hepatic lipid metabolism in nonlipodystrophic *Apoe* KO controls (Fig. 2, *A*–*F*).

### AT Alleviates Insulin Resistance in Lipodystrophic *Seipin/Apoe* dKO Mice

**Figure 3.**
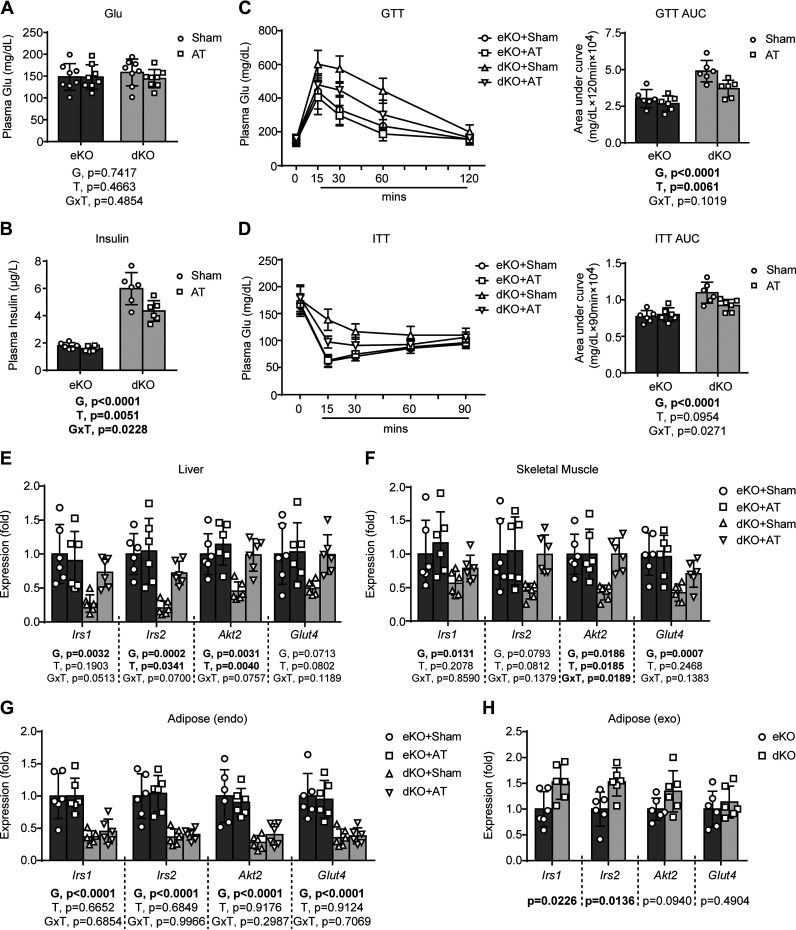
Adipose transplantation (AT) alleviates insulin resistance in lipodystrophic *Seipin/Apoe* double knockout (dKO) mice. *A*: plasma glucose concentration. *B*: plasma insulin concentration. *C*: glucose tolerance test (GTT) and quantitation of area under the curve (AUC). *D*: insulin tolerance test (ITT) and quantitation of AUC. *E*: insulin sensitivity-associated gene expression of the liver. *F*: insulin sensitivity-associated gene expression of the skeletal muscle. *G*: insulin sensitivity-associated gene expression of the endogenous (endo) subcutaneous adipose. *H*: insulin sensitivity-associated gene expression of the implanted exogenous (exo) adipose. For *H*, Student’s *t* tests are used to determine statistical significance, and the *P* values are displayed below the graph; for for *A*–*G*, the effects of genotype [G = dKO vs. *Apoe* knockout (eKO)] and treatment (T = AT vs. Sham) and G × T interactions were evaluated by two-way ANOVA followed by Tukey’s post hoc analysis, and the *P* values for the main and interaction effects are displayed below each graph. Error bars represent SD; *n* = 6–8 per group.

Insulin resistance is another core metabolic disorder in lipodystrophy-associated metabolic syndrome. Here, we demonstrated that lipodystrophic *Seipin/Apoe* dKO mice had similar plasma glucose levels but significantly increased insulin levels, compared to nonlipodystrophic *Apoe* KO controls; AT had no significant effect on plasma glucose levels, but markedly decreased plasma insulin levels in lipodystrophic *Seipin/Apoe* dKO mice (Fig. 3, *A* and *B*). Data from GTT and ITT suggested that AT corrected lipodystrophy-induced delay of glucose clearance in *Seipin/Apoe* dKO mice, indicating an improvement in both glucose and insulin resistance (Fig. 3, *C* and *D*). Moreover, AT also partially reversed lipodystrophy-induced inhibition of insulin sensitivity-associated gene expression in both the livers (*Irs2 and Akt2*) and the skeletal muscles (*Akt2*) of *Seipin/Apoe* dKO mice (Fig. 3, *E* and *F*). Interestingly, AT did not affect the expression of insulin sensitivity-associated genes in the endogenous adipose tissues of the recipient mice (Fig. 3*G*); however, compared to the transplanted exogenous donor fats in nonlipodystrophic *Apoe* KO, those transplanted into lipodystrophic *Seipin/Apoe* dKO mice exhibited higher expression of insulin sensitivity-associated genes (*Irs1* and *Irs2*) (Fig. 3*H*).

### AT Alleviates Atherosclerosis in Lipodystrophic *Seipin/Apoe* dKO Mice

**Figure 4.**
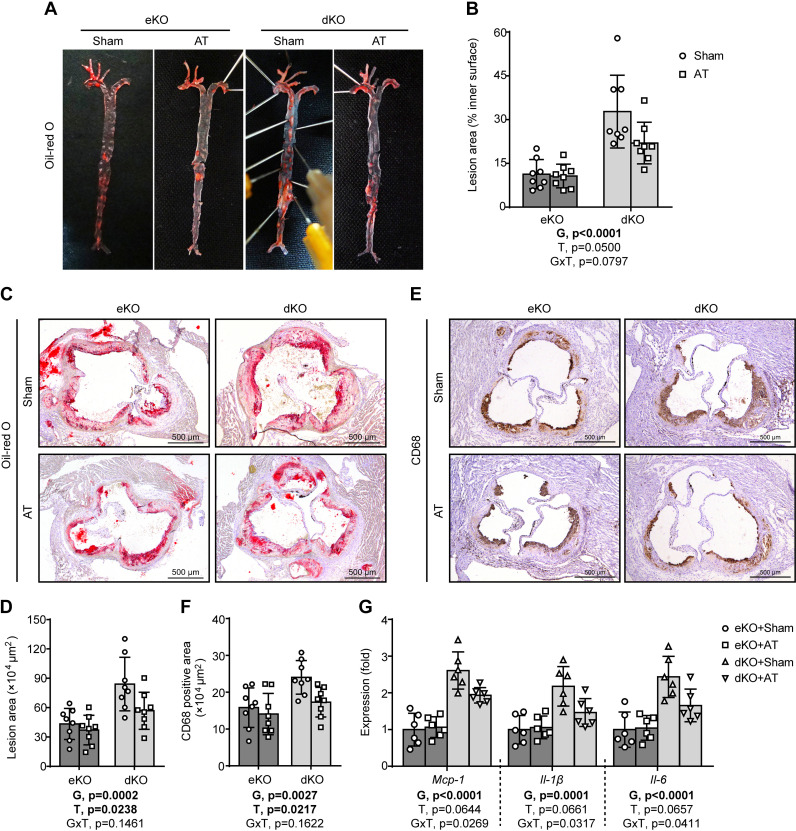
Adipose transplantation (AT) alleviates atherosclerosis in lipodystrophic *Seipin/Apoe* double knockout (dKO) mice. *A* and *B*: representative Oil-red O staining of the en face aorta (*A*) and quantitation of Oil-red O-positive area (*B*). *C* and *D*: representative Oil-red O staining of the aortic root cryosections (*C*) and quantitation of lesion area (*D*). *E* and *F*: representative CD68 immunochemical staining of the aortic root cryosections (*E*) and quantitation of CD68 positive area (*F*). *G*: aortic proinflammatory gene expression. The effects of genotype [G = dKO vs. *Apoe* knockout (eKO)] and treatment (T = AT vs. Sham) and G × T interactions were evaluated by two-way ANOVA followed by Tukey’s post hoc analysis. The *P* values for the main and interaction effects are displayed below each graph. Error bars represent SD; *n* = 6–8 per group.

Oil-red O staining showed that AT prevented the lipodystrophy-associated increase of atherosclerotic burden on the inner surface of the entire aorta in *Seipin/Apoe* dKO mice (Fig. 4, *A* and *B*). AT also ameliorated lipodystrophy-associated increase of atherosclerotic burden in the aortic root of *Seipin/Apoe* dKO mice (Fig. 4, *C* and *D*). Furthermore, AT inhibited lipodystrophy-associated increase of plaque macrophage infiltration and aortic expression of proinflammatory cytokines (*Mcp-1*, *Il-1β*, and *Il-6*) in *Seipin/Apoe* dKO mice, as shown by CD68 immunohistochemical staining (Fig. 4, *E* and *F*) and real-time quantitative PCR analysis (Fig. 4*G*), respectively. Conversely, AT had no significant effect on atherosclerosis in nonlipodystrophic *Apoe* KO controls (Fig. 4, *A*–*G*).

### AT Does Not Improve Perivascular Adipose Tissue Abnormality in Lipodystrophic *Seipin/Apoe* dKO Mice

**Figure 5.**
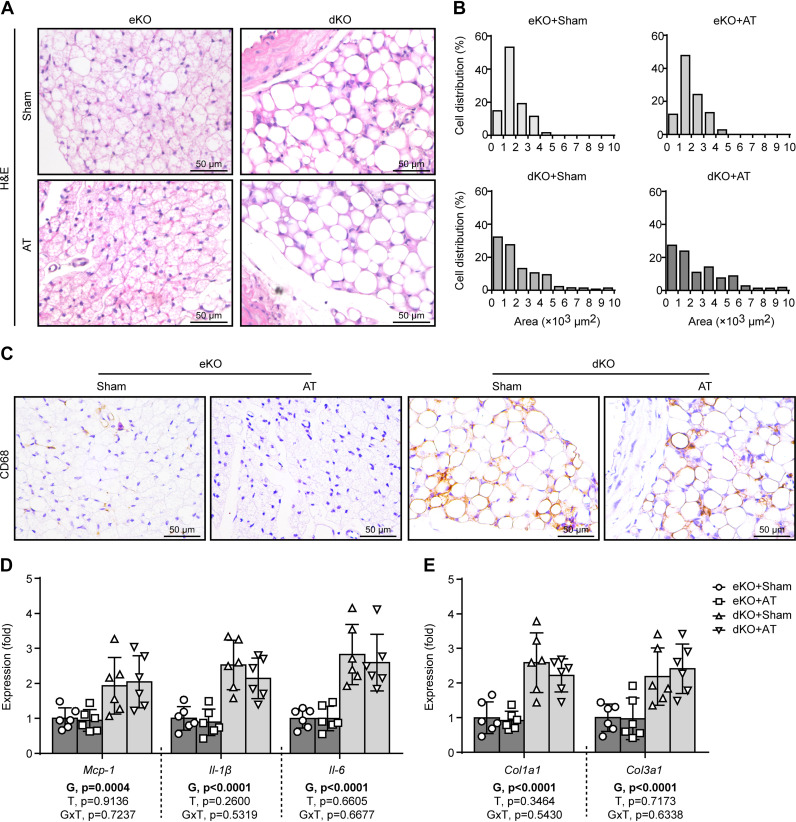
Adipose transplantation (AT) is incapable of correcting perivascular adipose tissue (PVAT) abnormality in lipodystrophic *Seipin/Apoe* double knockout (dKO) mice. *A*: representative hematoxylin and eosin (H&E) staining of the PVAT sections. *B*: quantitation of the size of perivascular adipocytes. *C*: CD68 immunochemical staining of the PVAT sections. *D*: PVAT proinflammatory gene expression. *E*: PVAT collagen-associated gene expression. The effects of genotype [G = dKO vs. *Apoe* knockout (eKO)] and treatment (T = AT vs. Sham) and G × T interactions were evaluated by two-way ANOVA followed by Tukey’s post hoc analysis. The *P* values for the main and interaction effects are displayed below each graph. Error bars represent SD; *n* = 6–8 per group.

Perivascular adipose tissue (PVAT) is increasingly acknowledged as a pivotal player in maintaining vascular homeostasis (27, 28). Our previous research has demonstrated that the PVAT of lipodystrophic *Seipin* KO mice is characterized by the presence of both large unilocular vacuoles and small adipocytes containing brightly eosinophilic cytoplasm, accumulation of proinflammatory macrophages, and increased fibrosis (9). Using H&E staining, we here showed that AT had no significant impact on the gross morphology of the PVAT (Fig. 5*A*) or the size distribution of the perivascular adipocytes (Fig. 5*B*) of lipodystrophic *Seipin/Apoe* dKO mice. Moreover, AT did not reduce the accumulation of CD68-positive macrophages in the PVAT of lipodystrophic *Seipin/Apoe* dKO mice (Fig. 5*C*), along with no reduction of the expression of macrophage-derived proinflammatory genes (*Mcp-1*, *Il-1β*, and *Il-6*) in the PVAT (Fig. 5*D*). Additionally, AT did not reduce PVAT fibrosis, as the expression of collagen-associated genes (*Col1a1* and *Col3a1*) in the PVAT of adipose-transplanted *Seipin/Apoe* dKO mice was no different from that of nontransplanted *Seipin/Apoe* dKO mice (Fig. 5*E*). Similarly, AT had no significant impact on the PVAT histology or gene expression in nonlipodystrophic *Apoe* KO controls (Fig. 5, *A–E*).

### AT Does Not Improve Vascular Dysfunction in Lipodystrophic *Seipin/Apoe* dKO Mice

**Figure 6.**
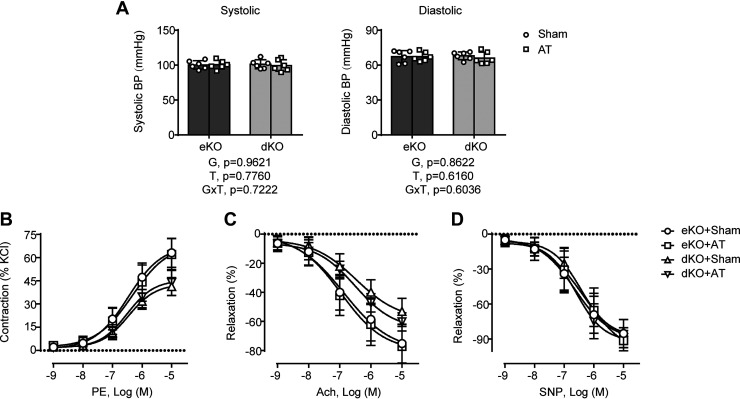
Adipose transplantation (AT) is incapable of improving vascular dysfunction in lipodystrophic *Seipin/Apoe* double knockout (dKO) mice. *A*: systolic (*left*) and diastolic (*right*) blood pressure. *B*: phenylephrine-induced contraction response. *C*: acetylcholine (Ach)-induced endothelial-dependent relaxation response. *D*: sodium nitroprusside (SNP)-induced endothelium-independent relaxation response. The effects of genotype [G = dKO vs. *Apoe* knockout (eKO)] and treatment (T = AT vs. Sham) and G × T interactions were evaluated by two-way ANOVA followed by Tukey’s post hoc analysis. The *P* values for the main and interaction effects are displayed below each graph. Error bars represent SD; *n* = 6–8 per group.

PVAT abnormailty has been demonstrated to impair vasoconstriction and relaxation in lipodystrophic *Seipin* KO mice (9); however, whether AT could correct lipodystrophy-related vascular dysfunction independent of PVAT is unclear. Here we demonstrated that AT had no significant effect on neither diastolic nor systolic blood pressure of lipodystrophic *Seipin/Apoe* dKO mice (Fig. 6*A*). Moreover, AT did not improve the PE-dependent aortic constriction (Fig. 6*B*) as well as the Ach-dependent endothelial relaxation (Fig. 6*C*) or SNP-dependent smooth muscle relaxation (Fig. 6*D*) in lipodystrophic *Seipin/Apoe* dKO mice. Likewise, AT had a null impact on vascular function in nonlipodystrophic *Apoe* KO controls (Fig. 6, *A*–*D*).

## DISCUSSION

In this study, we explored the effect of AT on lipodystrophy-associated metabolic cardiovascular diseases with a focus on vasculopathy in atherosclerosis-prone *Seipin/Apoe* dKO mice with normal rodent chow diet feeding. We demonstrated that (1) AT did not improve primary lipodystrophy, but could partially restore adipose function; (2) AT remarkably alleviated lipodystrophy-associated hyperlipidemia, hepatic steatosis, and insulin resistance; (3) AT significantly alleviated lipodystrophy-associated atherosclerosis; (4) AT, however, was incapable of improving lipodystrophy-associated PVAT abnormality or vascular dysfunction.

AT was first introduced in clinical medication in 1893 when fat collected from the arm was in a pioneering transplant that filled facial depressions caused by tuberculosis (29). Since then, AT has been widely used as a reconstructive surgery to improve appearance. In recent decades, its aim has been extended to understanding adipose biology and potentially treating metabolic diseases (29). In fact, experimental AT performed in rodents has already shown metabolic benefits in lipodystrophy. For instance, in lipodystrophic A-ZIP/F-1 mice, AT significantly reduces hyperglycemia and insulin resistance (30, 31); in lipodystrophic *Seipin* KO mice, AT also remarkably reverses dyslipidemia, hepatic steatosis, and insulin resistance (19, 20). However, whether AT could improve lipodystrophy-associated cardiovascular consequences remains to be elucidated. Here, using atherosclerosis-prone *Seipin/Apoe* dKO mice as a lipodystrophic mouse model, we showed that AT partially restored adipose function and markedly improved lipodystrophy-associated metabolic disorders, including hyperlipidemia, hepatic steatosis, and insulin resistance, followed by subsequently reduced atherosclerosis, although no significant improvement of PVAT abnormality or vascular dysfunction was observed. Our data therefore provide preliminary experimental evidence on the therapeutic potential of AT on lipodystrophy-associated metabolic cardiovascular diseases. The metabolic and vascular benefits offered by AT to the lipodystrophic *Seipin/Apoe* dKO mice were probably attributed to the functionality of the implanted exogenous fat grafts, rather than the improvement of primary lipodystrophy of the recipient *Seipin/Apoe* dKO mice, as the volume and gene expression of the endogenous adipose were not significantly changed after AT. Of note, transplantation of adipose-derived stem cells (ASCs) is now attracting increasing interest. Compared to mature adipocytes, ASCs are able to self-renew and differentiate into both white and brown adipocytes, as well as other cell lineages, such as endothelial cells and myocytes (32). Therefore, ASC transplantation may represent a promising option to reshape the primary lipodystrophic fat pads with potentially enhanced efficacy to rebuild metabolic homeostasis and prevent increased risks of developing metabolic cardiovascular diseases in lipodystrophy, which however needs further investigations.

Previously, we and others have depicted the metabolic phenotypes of lipodystrophic *Seipin* KO mice (21, 23, 33–35). These mice recapitulate most of the metabolic manifestations in human lipodystrophy caused by SEIPIN loss-of-function mutations, such as a complete loss of functional WAT, early onset of hepatic steatosis, and insulin resistance (36). However, in contrast to SEIPIN-deficient individuals who are generally hypertriglyceridemic, *Seipin* KO mice present a fasting hypotriglyceridemia due to insufficient adipose triglyceride mobilization and increased hepatic clearance of triglyceride-rich lipoproteins (21, 23, 33–35), AT is demonstrated to effectively correct hypotriglyceridemia but has no significant impact on plasma cholesterol levels in *Seipin* KO mice (20). Meanwhile, in atherosclerosis-prone low-density lipoprotein receptor (*Ldlr*) or *Apoe* KO mice, *Seipin* ablation causes combined hypertriglyceridemia and hypercholesterolemia and aggravates atherosclerosis (14, 15). AT, as shown in the current study, markedly ameliorates hypertriglyceridemia and hypercholesterolemia in *Seipin/Apoe* dKO mice, accompanied by significantly reduced atherosclerosis. The underlying mechanisms for the different lipid profiles as well as the different impacts of AT on the metabolic phenotypes in *Seipin* KO mice and atherosclerosis-prone *Seipin/Ldlr* dKO and *Seipin/Apoe* dKO mice are still not clear and need to be elucidated in future. Of note, in addition to genetic background-associated variation, the metabolic and cardiovascular phenotypes in lipodystrophic *Seipin* deficiency also show sex-specific differences in mice. For example, female *Seipin/Apoe* dKO mice preserve more adipose (especially BAT) than male mice (15). Moreover, plasma lipid levels of female *Seipin/Apoe* dKO mice are not as high as those of the males, and the severity of hepatic steatosis and atherogenesis in female mice are also significantly milder than their male counterparts (15). Whether estrogen contributes to the sex-specific variance as well as the interpretation of AT on the disease phenotypes is unclear. Therefore, to exclude the potential impact of estrogen, only male mice were selected in the current study.

Ectopic lipid accumulation due to a limited storage capacity in insufficient adiposity is traditionally known as the fundamental etiology for lipodystrophy-associated metabolic syndromes (6). Increasing evidence, however, suggests that adipose endocrine dysregulation is also playing a pivotal role in the process (6). Leptin and adiponectin are two pleiotropic hormones predominantly secreted by the adipose tissues (37). The levels of these two adipokines are closely associated with the volume and function of the adipose and thus are frequently used as biomarkers for the diagnosis and phenotyping of lipodystrophy (37). In lipodystrophic *Seipin* KO mice, both of these two adipokines are dramatically reduced (19–21). AT was found capable of restoring plasma leptin levels in lipodystrophic *Seipin/Apoe* dKO mice but had no significant effect on adiponectin concentration, likewise in *Seipin* KO mice subjected to AT (19, 20). The underlying mechanisms of the inconsistent impacts of AT on leptin and adiponectin secretion are still not defined and need to be elucidated further. Interestingly, leptin administration has been shown to favorably affect the metabolic profile, including dyslipidemia, hepatic steatosis, and insulin resistance, in both lipodystrophic patients (38–42) and mice (19, 43–47), suggesting that the therapeutic potential of AT in rescuing lipodystrophy-associated metabolic derangements may at least partially depend on proper leptin signaling. Future investigations might focus specifically on the effects of leptin supplementation as well as transplantation of leptin-deficient adipose tissues from *ob/ob* mice to confirm the contribution of leptin signaling in lipodystrophy-associated atherosclerosis in *Seipin/Apoe* dKO mice. In addition to leptin administration, adiponectin supplementation has also shown therapeutic potential in mouse studies (48). In lipodystrophic peroxisome proliferator-activated receptor-γ (PPARγ) heterozygous mice with additional pharmacological inhibition of PPARγ/retinoid-X receptor (RXR) activity, adiponectin supplementation decreases insulin resistance by increasing fatty acid β-oxidation and reducing muscular and hepatic triglyceride accumulation (49); moreover, in a mouse model of HAART-associated lipodystrophy syndrome, adiponectin replacement therapy also markedly ameliorates ritonavir-induced increases of plasma triglyceride and free fatty acids levels (50). Notably, leptin or adiponectin therapy alone cannot fully reverse lipodystrophy-associated metabolic abnormalities (6, 51), which might translate into a limited impact on cardiovascular phenotypes. As reflected in the current study, AT only partially inhibits lipodystrophy-associated atherosclerosis with an almost null effect on lipodystrophy-associated vascular contraction or relaxation dysfunction in *Seipin/Apoe* dKO mice. Whether it is attributed to the inability of AT to restore adiponectin secretion in lipodystrophic *Seipin/Apoe* dKO mice is unclear. Further explorations might also consider a combination of leptin and adiponectin administration in rescuing lipodystrophy-associated metabolic cardiovascular diseases in *Seipin/Apoe* dKO mice.

Apart from leptin and adiponectin secretion, the adipose tissue also profoundly influences systemic metabolism and cardiovascular health by regulating thermogenesis in adaption to changes in ambient temperatures. BAT is traditionally known as the core thermoregulatory organ in rodents and human infants (52, 53). Recently, it was also found in adult humans (52, 53). WAT can also contribute to thermogenesis by transforming into beige/brite adipose under cold exposure and other stresses such as sympathetic nervous system activation (54). In lipodystrophic mice, such as *Seipin* KO mice, the thermogenic biology is remarkably altered due to the loss of functional adipose (55). Whether AT could reshape thermogenesis to improve lipodystrophy-associated metabolic disorders and cardiovascular consequences is still unclear and not investigated in the current study. It may also be worth mentioning that mice and humans differ in their thermal physiology (56). One prominent dissimilarity is that humans are often living close to their thermoneutral zone, a term used to define the ambient temperatures where the metabolic rate is minimal and constant (57); therefore, the thermoregulation is toward heat dissipation rather than generation; in contrast, mice are typically housed in room temperatures (20–22°C) that significantly fall below their thermoneutral zone (29–32°C) (56). Such room temperature housing, as used in the current study, causes significant thermal stress to mice and about half of their energy expenditure has to be devoted to maintaining their core body temperature (56). A growing body of evidence has demonstrated that housing mice at room temperature favors compensatory signaling pathways that significantly impede the phenotypic interpretation when studying metabolic cardiovascular diseases (54). Therefore, the presence of thermal stress in *Seipin/Apoe* dKO mice might limit the translation to humans.

In conclusion, our study demonstrates that AT effectively ameliorates hyperlipidemia, hepatic steatosis, insulin resistance, and atherosclerosis but has a null effect on PVAT abnormality or vascular dysfunction in lipodystrophic *Seipin/Apoe* dKO mice. The current study provides preliminary experimental evidence on the therapeutic potential of AT on lipodystrophy-associated metabolic cardiovascular diseases.

## DATA AVAILABILITY

Data will be made available upon reasonable request.

## GRANTS

This study was supported by the Basic Research Program of the Department of Education of Liaoning Province (JYTMS20230579 to J. Liao) and the National Natural Science Foundation of China (81900378 to M. Wang).

## DISCLOSURES 

No conflicts of interest, financial or otherwise, are declared by the authors.

## AUTHOR CONTRIBUTIONS

Z.M., J.L., and M.W. conceived and designed research; Z.M., C.L., M.X., Y.T., H.L., and X.W. performed experiments; Z.M. analyzed data; Z.M. interpreted results of experiments; Z.M. and M.W. prepared figures; Z.M. and M.W. drafted manuscript; J.L. edited and revised manuscript; C.L., M.X., Y.T., H.L., X.W., J.L., and M.W. approved final version of manuscript.

## References

[1] Sakers A, De Siqueira MK, Seale P, Villanueva CJ. Adipose-tissue plasticity in health and disease. Cell 185: 419–446, 2022. doi:10.1016/j.cell.2021.12.016. 35120662 PMC11152570

[2] Pilkington AC, Paz HA, Wankhade UD. Beige adipose tissue identification and marker specificity-overview. Front Endocrinol (Lausanne) 12: 599134, 2021. doi:10.3389/fendo.2021.599134. 33776911 PMC7996049

[3] Oikonomou EK, Antoniades C. The role of adipose tissue in cardiovascular health and disease. Nat Rev Cardiol 16: 83–99, 2019. doi:10.1038/s41569-018-0097-6. 30287946

[4] Koliaki C, Liatis S, Kokkinos A. Obesity and cardiovascular disease: revisiting an old relationship. Metabolism 92: 98–107, 2019. doi:10.1016/j.metabol.2018.10.011. 30399375

[5] Longo M, Zatterale F, Naderi J, Parrillo L, Formisano P, Raciti GA, Beguinot F, Miele C. Adipose tissue dysfunction as determinant of obesity-associated metabolic complications. Int J Mol Sci 20: 2358, 2019. doi:10.3390/ijms20092358. 31085992 PMC6539070

[6] Fiorenza CG, Chou SH, Mantzoros CS. Lipodystrophy: pathophysiology and advances in treatment. Nat Rev Endocrinol 7: 137–150, 2011. doi:10.1038/nrendo.2010.199. 21079616 PMC3150735

[7] Magré J, Delépine M, Khallouf E, Gedde-Dahl T Jr, Van Maldergem L, Sobel E, , et al Identification of the gene altered in Berardinelli-Seip congenital lipodystrophy on chromosome 11q13. Nat Genet 28: 365–370, 2001. doi:10.1038/ng585. 11479539

[8] Rao MJ, Goodman JM. Seipin: harvesting fat and keeping adipocytes healthy. Trends Cell Biol 31: 912–923, 2021. doi:10.1016/j.tcb.2021.06.003. 34215489 PMC8526392

[9] Wang M, Xing J, Liu M, Gao M, Liu Y, Li X, Hu L, Zhao X, Liao J, Liu G, Dong J. Deletion of Seipin attenuates vascular function and the anticontractile effect of perivascular adipose tissue. Front Cardiovasc Med 8: 706924, 2021. doi:10.3389/fcvm.2021.706924. 34409079 PMC8365033

[10] Szymanski KM, Binns D, Bartz R, Grishin NV, Li WP, Agarwal AK, Garg A, Anderson RG, Goodman JM. The lipodystrophy protein seipin is found at endoplasmic reticulum lipid droplet junctions and is important for droplet morphology. Proc Natl Acad Sci U S A 104: 20890–20895, 2007. doi:10.1073/pnas.0704154104. 18093937 PMC2409237

[11] Payne VA, Grimsey N, Tuthill A, Virtue S, Gray SL, Dalla Nora E, Semple RK, O'Rahilly S, Rochford JJ. The human lipodystrophy gene BSCL2/seipin may be essential for normal adipocyte differentiation. Diabetes 57: 2055–2060, 2008. doi:10.2337/db08-0184. 18458148 PMC2494687

[12] Fei W, Shui G, Gaeta B, Du X, Kuerschner L, Li P, Brown AJ, Wenk MR, Parton RG, Yang H. Fld1p, a functional homologue of human seipin, regulates the size of lipid droplets in yeast. J Cell Biol 180: 473–482, 2008. doi:10.1083/jcb.200711136. 18250201 PMC2234226

[13] Chen W, Yechoor VK, Chang BH, Li MV, March KL, Chan L. The human lipodystrophy gene product Berardinelli-Seip congenital lipodystrophy 2/seipin plays a key role in adipocyte differentiation. Endocrinology 150: 4552–4561, 2009. doi:10.1210/en.2009-0236. 19574402 PMC2754678

[14] Wang M, Gao M, Liao J, Qi Y, Du X, Wang Y, Li L, Liu G, Yang H. Adipose tissue deficiency results in severe hyperlipidemia and atherosclerosis in the low-density lipoprotein receptor knockout mice. Biochim Biophys Acta 1861: 410–418, 2016. doi:10.1016/j.bbalip.2016.02.018. 26921684

[15] Liao J, Liu X, Gao M, Wang M, Wang Y, Wang F, Huang W, Liu G. Dyslipidemia, steatohepatitis and atherogenesis in lipodystrophic apoE deficient mice with Seipin deletion. Gene 648: 82–88, 2018. doi:10.1016/j.gene.2018.01.062. 29428127

[16] Joubert M, Jagu B, Montaigne D, Marechal X, Tesse A, Ayer A, Dollet L, Le May C, Toumaniantz G, Manrique A, Charpentier F, Staels B, Magré J, Cariou B, Prieur X. The sodium-glucose cotransporter 2 inhibitor dapagliflozin prevents cardiomyopathy in a diabetic lipodystrophic mouse model. Diabetes 66: 1030–1040, 2017. doi:10.2337/db16-0733. 28052965

[17] Bai B, Yang W, Fu Y, Foon HL, Tay WT, Yang K, Luo C, Gunaratne J, Lee P, Zile MR, Xu A, Chin CW, Lam CS, Han W, Wang Y. Seipin knockout mice develop heart failure with preserved ejection fraction. JACC Basic Transl Sci 4: 924–937, 2019. doi:10.1016/j.jacbts.2019.07.008. 31909301 PMC6939002

[18] Wu X, Liu X, Wang H, Zhou Z, Yang C, Li Z, Zhang Y, Shi X, Zhang L, Wang Y, Xian X, Liu G, Huang W. Seipin deficiency accelerates heart failure due to calcium handling abnormalities and endoplasmic reticulum stress in mice. Front Cardiovasc Med 8: 644128, 2021. doi:10.3389/fcvm.2021.644128. 33778025 PMC7990891

[19] Liu XJ, Wu XY, Wang H, Wang SX, Kong W, Zhang L, Liu G, Huang W. Renal injury in Seipin-deficient lipodystrophic mice and its reversal by adipose tissue transplantation or leptin administration alone: adipose tissue-kidney crosstalk. FASEB J 32: 5550–5562, 2018. doi:10.1096/fj.201701427R. 29738274

[20] Wang H, Xu PF, Li JY, Liu XJ, Wu XY, Xu F, Xie BC, Huang XM, Zhou ZH, Kayoumu A, Liu G, Huang W. Adipose tissue transplantation ameliorates lipodystrophy-associated metabolic disorders in seipin-deficient mice. Am J Physiol Endocrinol Metab 316: E54–E62, 2019. doi:10.1152/ajpendo.00180.2018. 30457912

[21] Cui X, Wang Y, Tang Y, Liu Y, Zhao L, Deng J, Xu G, Peng X, Ju S, Liu G, Yang H. Seipin ablation in mice results in severe generalized lipodystrophy. Hum Mol Genet 20: 3022–3030, 2011. doi:10.1093/hmg/ddr205. 21551454

[22] Deng Y, Li Z, An X, Fan R, Wang Y, Li J, Yang X, Liao J, Xia Y. Hyperhomocysteinemia promotes cardiac hypertrophy in hypertension. Oxid Med Cell Longev 2022: 1486157, 2022. doi:10.1155/2022/1486157. 36046692 PMC9423973

[23] Gao M, Wang M, Guo X, Qiu X, Liu L, Liao J, Liu J, Lu G, Wang Y, Liu G. Expression of seipin in adipose tissue rescues lipodystrophy, hepatic steatosis and insulin resistance in seipin null mice. Biochem Biophys Res Commun 460: 143–150, 2015. doi:10.1016/j.bbrc.2015.02.147. 25757906

[24] Liu L, Liang C, Wang X, Ding X, Lu Y, Dong J, Han M, Yang H, Gao M, Liao J. Surgical fat removal exacerbates metabolic disorders but not atherogenesis in LDLR^−/−^ mice fed on high-fat diet. Sci Rep 10: 2181, 2019. doi:10.1038/s41598-019-54392-8. 31780791 PMC6883051

[25] Liu L, Liang CX, Wang XW, Pei KX, Ma XD, Zhang CX, Dong JH, Gao MM, Liao JW. Preliminary adipose removal did not prevent diet-induced metabolic disorders in mice. Chin Med J (Engl) 134: 716–724, 2021. doi:10.1097/CM9.0000000000001334. 33410621 PMC7989994

[26] Liao J, Xie Y, Lin Q, Yang X, An X, Xia Y, Du J, Wang F, Li HH. Immunoproteasome subunit beta5i regulates diet-induced atherosclerosis through altering MERTK-mediated efferocytosis in Apoe knockout mice. J Pathol 250: 275–287, 2020. doi:10.1002/path.5368. 31758542

[27] Kim HW, Shi H, Winkler MA, Lee R, Weintraub NL. Perivascular adipose tissue and vascular perturbation/atherosclerosis. Arterioscler Thromb Vasc Biol 40: 2569–2576, 2020. doi:10.1161/ATVBAHA.120.312470. 32878476 PMC7577939

[28] Hu H, Garcia-Barrio M, Jiang ZS, Chen YE, Chang L. Roles of perivascular adipose tissue in hypertension and atherosclerosis. Antioxid Redox Signal 34: 736–749, 2021. doi:10.1089/ars.2020.8103. 32390459 PMC7910418

[29] Tran TT, Kahn CR. Transplantation of adipose tissue and stem cells: role in metabolism and disease. Nat Rev Endocrinol 6: 195–213, 2010. doi:10.1038/nrendo.2010.20. 20195269 PMC4362513

[30] Gavrilova O, Marcus-Samuels B, Graham D, Kim JK, Shulman GI, Castle AL, Vinson C, Eckhaus M, Reitman ML. Surgical implantation of adipose tissue reverses diabetes in lipoatrophic mice. J Clin Invest 105: 271–278, 2000. doi:10.1172/JCI7901. 10675352 PMC377444

[31] Kim JK, Gavrilova O, Chen Y, Reitman ML, Shulman GI. Mechanism of insulin resistance in A-ZIP/F-1 fatless mice. J Biol Chem 275: 8456–8460, 2000. doi:10.1074/jbc.275.12.8456. 10722680

[32] Mizuno H, Tobita M, Uysal AC. Concise review: adipose-derived stem cells as a novel tool for future regenerative medicine. Stem cells (Cells 30: 804–810, 2012. doi:10.1002/stem.1076. 22415904

[33] Chen W, Chang B, Saha P, Hartig SM, Li L, Reddy VT, Yang Y, Yechoor V, Mancini MA, Chan L. Berardinelli-seip congenital lipodystrophy 2/seipin is a cell-autonomous regulator of lipolysis essential for adipocyte differentiation. Mol Cell Biol 32: 1099–1111, 2012. doi:10.1128/MCB.06465-11. 22269949 PMC3295006

[34] Prieur X, Dollet L, Takahashi M, Nemani M, Pillot B, Le May C, Mounier C, Takigawa-Imamura H, Zelenika D, Matsuda F, Fève B, Capeau J, Lathrop M, Costet P, Cariou B, Magré J. Thiazolidinediones partially reverse the metabolic disturbances observed in Bscl2/seipin-deficient mice. Diabetologia 56: 1813–1825, 2013. doi:10.1007/s00125-013-2926-9. 23680914

[35] Wang M, Gao M, Liao J, Han Y, Wang Y, Liu G. Dysfunction of lipid metabolism in lipodystrophic Seipin-deficient mice. Biochem Biophys Res Commun 461: 206–210, 2015. doi:10.1016/j.bbrc.2015.03.117. 25866184

[36] Li Y, Yang X, Peng L, Xia Q, Zhang Y, Huang W, Liu T, Jia D. Role of Seipin in human diseases and experimental animal models. Biomolecules 12: 840, 2022. doi:10.3390/biom12060840. 35740965 PMC9221541

[37] Zhao S, Kusminski CM, Scherer PE. Adiponectin, leptin and cardiovascular disorders. Circ Res 128: 136–149, 2021. doi:10.1161/CIRCRESAHA.120.314458. 33411633 PMC7799441

[38] Oral EA, Simha V, Ruiz E, Andewelt A, Premkumar A, Snell P, Wagner AJ, DePaoli AM, Reitman ML, Taylor SI, Gorden P, Garg A. Leptin-replacement therapy for lipodystrophy. N Engl J Med 346: 570–578, 2002. doi:10.1056/NEJMoa012437. 11856796

[39] Javor ED, Ghany MG, Cochran EK, Oral EA, DePaoli AM, Premkumar A, Kleiner DE, Gorden P. Leptin reverses nonalcoholic steatohepatitis in patients with severe lipodystrophy. Hepatology 41: 753–760, 2005. doi:10.1002/hep.20672. 15791619

[40] Javor ED, Cochran EK, Musso C, Young JR, Depaoli AM, Gorden P. Long-term efficacy of leptin replacement in patients with generalized lipodystrophy. Diabetes 54: 1994–2002, 2005. doi:10.2337/diabetes.54.7.1994. 15983199

[41] Ebihara K, Kusakabe T, Hirata M, Masuzaki H, Miyanaga F, Kobayashi N, Tanaka T, Chusho H, Miyazawa T, Hayashi T, Hosoda K, Ogawa Y, DePaoli AM, Fukushima M, Nakao K. Efficacy and safety of leptin-replacement therapy and possible mechanisms of leptin actions in patients with generalized lipodystrophy. J Clin Endocrinol Metab 92: 532–541, 2007. doi:10.1210/jc.2006-1546. 17118991

[42] Mosbah H, Vantyghem MC, Nobécourt E, Andreelli F, Archambeaud F, Bismuth E, Briet C, Cartigny M, Chevalier B, Donadille B, Daguenel A, Fichet M, Gautier JF, Janmaat S, Jéru I, Legagneur C, Leguier L, Maitre J, Mongeois E, Poitou C, Renard E, Reznik Y, Spiteri A, Travert F, Vergès B, Zammouri J, Vigouroux C, Vatier C. Therapeutic indications and metabolic effects of metreleptin in patients with lipodystrophy syndromes: real-life experience from a national reference network. Diabetes Obes Metab 24: 1565–1577, 2022. doi:10.1111/dom.14726. 35445532 PMC9541305

[43] Shimomura I, Hammer RE, Ikemoto S, Brown MS, Goldstein JL. Leptin reverses insulin resistance and diabetes mellitus in mice with congenital lipodystrophy. Nature 401: 73–76, 1999. doi:10.1038/43448. 10485707

[44] Ebihara K, Ogawa Y, Masuzaki H, Shintani M, Miyanaga F, Aizawa-Abe M, Hayashi T, Hosoda K, Inoue G, Yoshimasa Y, Gavrilova O, Reitman ML, Nakao K. Transgenic overexpression of leptin rescues insulin resistance and diabetes in a mouse model of lipoatrophic diabetes. Diabetes 50: 1440–1448, 2001. doi:10.2337/diabetes.50.6.1440. 11375346

[45] Colombo C, Cutson JJ, Yamauchi T, Vinson C, Kadowaki T, Gavrilova O, Reitman ML. Transplantation of adipose tissue lacking leptin is unable to reverse the metabolic abnormalities associated with lipoatrophy. Diabetes 51: 2727–2733, 2002. doi:10.2337/diabetes.51.9.2727. 12196465

[46] Asilmaz E, Cohen P, Miyazaki M, Dobrzyn P, Ueki K, Fayzikhodjaeva G, Soukas AA, Kahn CR, Ntambi JM, Socci ND, Friedman JM. Site and mechanism of leptin action in a rodent form of congenital lipodystrophy. J Clin Invest 113: 414–424, 2004. doi:10.1172/JCI200419511. 14755338 PMC324539

[47] Cortés VA, Cautivo KM, Rong S, Garg A, Horton JD, Agarwal AK. Leptin ameliorates insulin resistance and hepatic steatosis in Agpat2−/− lipodystrophic mice independent of hepatocyte leptin receptors. J Lipid Res 55: 276–288, 2014. doi:10.1194/jlr.M045799. 24293639 PMC3886666

[48] Duntas LH, Popovic V, Panotopoulos G. Adiponectin: novelties in metabolism and hormonal regulation. Nutr Neurosci 7: 195–200, 2004. doi:10.1080/10284150400009998. 15682645

[49] Yamauchi T, Kamon J, Waki H, Terauchi Y, Kubota N, Hara K, Mori Y, Ide T, Murakami K, Tsuboyama-Kasaoka N, Ezaki O, Akanuma Y, Gavrilova O, Vinson C, Reitman ML, Kagechika H, Shudo K, Yoda M, Nakano Y, Tobe K, Nagai R, Kimura S, Tomita M, Froguel P, Kadowaki T. The fat-derived hormone adiponectin reverses insulin resistance associated with both lipoatrophy and obesity. Nat Med 7: 941–946, 2001. doi:10.1038/90984. 11479627

[50] Xu A, Yin S, Wong L, Chan KW, Lam KS. Adiponectin ameliorates dyslipidemia induced by the human immunodeficiency virus protease inhibitor ritonavir in mice. Endocrinology 145: 487–494, 2004. doi:10.1210/en.2003-1140. 14592951

[51] Polyzos SA, Perakakis N, Mantzoros CS. Fatty liver in lipodystrophy: a review with a focus on therapeutic perspectives of adiponectin and/or leptin replacement. Metabolism 96: 66–82, 2019. doi:10.1016/j.metabol.2019.05.001. 31071311

[52] Jung SM, Sanchez-Gurmaches J, Guertin DA. Brown adipose tissue development and metabolism. Handb Exp Pharmacol 251: 3–36, 2019. doi:10.1007/164_2018_168. 30203328 PMC7330484

[53] Scheele C, Wolfrum C. Brown adipose crosstalk in tissue plasticity and human metabolism. Endocr Rev 41: 53–65, 2020. doi:10.1210/endrev/bnz007. 31638161 PMC7006230

[54] Lac M, Tavernier G, Moro C. Does housing temperature influence glucose regulation and muscle-fat crosstalk in mice? Biochimie 210: 35–39, 2023. doi:10.1016/j.biochi.2023.01.019. 36758717

[55] Zhou H, Xu C, Lee H, Yoon Y, Chen W. Berardinelli-Seip congenital lipodystrophy 2/SEIPIN determines brown adipose tissue maintenance and thermogenic programing. Mol Metab 36: 100971, 2020. doi:10.1016/j.molmet.2020.02.014. 32246911 PMC7136632

[56] Škop V, Guo J, Liu N, Xiao C, Hall KD, Gavrilova O, Reitman ML. Mouse thermoregulation: introducing the concept of the thermoneutral point. Cell Rep 31: 107501, 2020. doi:10.1016/j.celrep.2020.03.065. 32294435 PMC7243168

[57] Gordon CJ. The mouse thermoregulatory system: its impact on translating biomedical data to humans. Physiol Behav 179: 55–66, 2017. doi:10.1016/j.physbeh.2017.05.026. 28533176 PMC6196327

